# Field-scale evaluation of satellite-derived vegetation indices and image timing for in-season nitrogen management in corn

**DOI:** 10.3389/fpls.2026.1731400

**Published:** 2026-03-20

**Authors:** Ana Morales-Ona, Katsutoshi Mizuta, James Camberato, Robert Nielsen, Yuxin Miao, Davide Cammarano, German Mandrini, Daniel Quinn

**Affiliations:** 1 Department of Agronomy, Purdue University, West Lafayette, IN, United States; 2 Department of Plant and Soil Sciences, University of Kentucky, Lexington, KY, United States; 3 Precision Agriculture Center, University of Minnesota, Saint Paul, MN, United States; 4 Department of Agroecology, iClimate, CBIO, Aarhus University, Tjele, Denmark

**Keywords:** in-season nitrogen management, precision agriculture, remote sensing, satellite imagery, vegetation indices

## Abstract

**Introduction:**

Traditional methods to determine the agronomic optimum nitrogen rate (AONR) for corn (*Zea mays* L.) rely on grain yield data, limiting in-season decision-making for nitrogen (N) management. Vegetation indices (VIs) derived from satellite imagery can serve as proxies for grain yield and help estimate in-season AONR, enabling timely sidedress applications. This study aimed to (1) quantify how VI–yield relationships vary during the vegetative period across fields with different tillage systems and crop residue; (2) determine whether VI–N response curves can be used to estimate AONR (AONRvi); and (3) assess the accuracy of AONRvi relative to yield-based AONR (AONRy).

**Methods:**

Three rainfed on-farm field trials with contrasting tillage systems and four to five N rates were conducted in Indiana in 2021. PlanetScope imagery (3-m resolution) was used to calculate 16 VIs (8 NIR-based and 8 RGB-based) across multiple growth stages. Linear regressions between yield and VIs were used to assess strength of their relationship, followed by VI–N response curves to estimate AONRvi.

**Results and Discussion:**

During the vegetative period, VI–yield relationships were generally weak (R^2^ ≤ 0.31) and varied across fields, with fewer significant relationships under higher crop residue conditions. Of the VI–N response curves evaluated, 17% met selection criteria for estimating AONRvi, with lower proportions observed in higher-residue systems. Mid-vegetative period imagery (V10–V11) produced the smallest deviations from AONRy for NIR-based indices, although no single VI consistently performed best across fields and timings. These results indicate that 3-m satellite imagery has potential to detect crop N response under commercial field conditions, but its reliability for estimating in-season AONR depends on management context, image timing, and spectral domain.

## 1 Introduction

Nitrogen (N) is an important nutrient for corn (*Zea mays* L.) production, as it plays a central role in photosynthesis, biomass accumulation, and grain yield formation (Cassman et al., 2002). However, optimizing N application remains a persistent challenge due to the temporal and spatial variability of soil N availability and crop N demand. Inefficient N management leads to both economic losses and environmental concerns, including groundwater contamination, greenhouse gas emissions, and eutrophication of water bodies (Cameron et al., 2013; Chen et al., 2020; Sharma and Bali, 2018). Across the Midwest region of the United States of America, significant spatial variability exists in crop yield stability even within the same field. This variability affects N use efficiency (calculated as grain N removal divided by N applied), which can range from 48% in stable low-yielding areas to 88% in stable high-yielding areas (Basso et al., 2019). Traditional uniform N applications overlook this variability, whereas site-specific N management enables dynamic N adjustment in space and time based on crop physiological status (Doerge, 2002).

Remote sensing technologies offer tools for assessing crop N status and supporting site-specific applications. These technologies include proximal sensors (e.g., SPAD, GreenSeeker, Crop Circle) (Barker and Sawyer, 2010; Cilia et al., 2014; Daughtry et al., 2000; Maresma et al., 2018; Paiao et al., 2020; Quemada et al., 2014; Rambo et al., 2010; Vergara-Díaz et al., 2016; Viña et al., 2011), airborne platforms (Cilia et al., 2014; Maresma et al., 2018; Miao et al., 2009; Quemada et al., 2014; Scharf and Lory, 2002; Viña et al., 2011), unmanned aerial vehicles (UAVs) (Burns et al., 2022; Killeen et al., 2024; Oglesby et al., 2022; Sunoj et al., 2021) and satellite imagery (Bausch and Khosla, 2010). While proximal and UAV sensors provide high spatial resolution and greater sensitivity to canopy-level variation, they are limited in spatial scale and operational feasibility, making them less feasible for commercial on-farm applications. Satellite imagery present a more scalable alternative for precision N management, offering data collection over larger areas (Bausch and Khosla, 2010), though its coarser spatial resolution may reduce sensitivity to early-season variability and increase interference from soil and crop residue signals.

Vegetation indices (VIs) derived from remote sensing data serve as proxies for plant N status and grain yield, offering an objective and scalable means to inform N management decisions (Barzin et al., 2020; Kumar et al., 2023; Sunoj et al., 2021). Studies using UAV imagery have reported varying degrees of correlation between VIs and grain yield. For instance, Barzin et al. (2020) found that the Optimized Soil Adjusted Vegetation Index (OSAVI) explained approximately 63% of yield variability at the V3 growth stage, whereas Burns et al. (2022) reported lower coefficients of determination (R² up to 0.29) at V6. These differences highlight the influence of growth stage and environmental context when interpreting VI–yield relationships. Studies comparing satellite, UAV, and proximal sensors suggest that higher spatial resolution platforms (e.g., UAV and handheld chlorophyll meters) improve sensitivity to N stress, particularly during early vegetative stages when soil background effects are more pronounced (Daughtry et al., 2000; Jones et al., 2015; Maresma et al., 2018).

In addition to growth stage and sensor spatial resolution, agronomic management factors such as tillage system can affect VI reliability. Studies show that high surface residue in strip-till or no-till systems increases early-season reflectance, leading to VI saturation (Gausman et al., 1975; Jones et al., 2015). Likewise, water availability interacts with N uptake and canopy reflectance (Bennett et al., 1989). These interactions highlight the need to evaluate VI performance under realistic management and environmental variability.

Despite progress in high-spatial resolution UAV and proximal sensing research, less is known about how satellite-derived VIs perform under commercial on-farm conditions that capture the spatial heterogeneity and management complexity of real production systems. At the 3-m spatial resolution of PlanetScope imagery, individual pixels often contain mixtures of soil, crop residue, and canopy, particularly during early vegetative stages. Tillage and residue management therefore influence the spectral signal and may constrain the reliability and transferability of satellite-based VI for N management at the field-scale (Gausman et al., 1975; Psomiadis et al., 2016; Rocchini, 2007). More broadly, cross-domain variability arising from sensor differences, environmental conditions, and geographic context has been recognized as a key limitation in remote sensing applications (Li et al., 2025; Zheng et al., 2024). Evaluating VI performance across contrasting residue environments is essential to determine under which management contexts satellite-derived VI remain informative. Furthermore, while many studies emphasize yield prediction, fewer assess whether satellite-derived VIs can reliably estimate agronomic optimum nitrogen rates (AONR) during the vegetative period, when in-season N decisions are made.

This study addresses that gap by evaluating 3-m PlanetScope imagery under commercial field-scale conditions to quantity VI-yield relationships across contrasting tillage systems and to test whether VI–N response curves can support in-season AONR estimation. By integrating management context, image timing, and spectral domain, this work provides a field-scale assessment of the practical limits and potential of satellite imagery for operational precision N management. Specifically, the objectives of this study were to: (1) quantify how the relationship between satellite-derived VIs and corn grain yield varies during the vegetative period across fields differing in tillage and crop residue, and contrast these relationships with a reproductive-stage observation for comparison; (2) determine whether VI–N rate response curves can be used to estimate AONR (AONRvi) during the vegetative period; and (3) assess the accuracy of AONRvi relative to yield-based AONR (AONRy).

We hypothesized that: (H1) early-season VI–yield relationships would be weaker under strip-tillage due to greater soil–residue interference within 3-m satellite pixels compared to conventionally tilled fields; (H2) differences in VI–yield relationships across fields with different tillage systems would diminish at reproductive stages because near-complete canopy closure reduces the spectral influence of soil and crop residue; (H3) greater crop residue in strip-till systems would weaken the VI response to applied N during the vegetative period, resulting in fewer usable VI–N response curves for estimating AONRvi; and (H4) vegetative-stage images acquired closer to canopy closure would produce AONRvi values that most closely match AONRy compared to earlier stages. By evaluating VI–yield relationships, VI–N response curves, and AONR estimation under contrasting tillage systems at the commercial field scale, this study provides expectations for the use of 3-m satellite imagery in operational precision nitrogen management. This work identifies growth stages and spectral domains under which satellite-derived VIs are most informative in commercial farming operations, thereby strengthening their practical application in site-specific N management.

## 2 Materials and methods

### 2.1 Site description and field trials information

Three field trials were conducted in 2021. One was located at the Davis-Purdue Agricultural Center (40.245797°, -85.149480°, elevation 296 m above sea level) near Farmland, in east-central IN, and two at a commercial farm (40.564331°, -86.962348°, elevation 221 m above sea level) near Brookston, in west-central IN. Although the Davis-Purdue Agricultural Center is a university-operated research facility, the field used in this study is managed under standard commercial practices and is specifically designated for field-scale agronomic research. As such, all field trials reflect typical farming conditions. Soil information for each field trial is detailed in **Supplementary Table 1**, and the experiment area, planting dates, hybrids, seeding rates, previous crop, tillage practices, and field trial acronyms are given in **Table 1**. Hereafter, the three field trials are referred to by their acronyms, which indicate the tillage practice (ST for strip-till, CT for conventional tillage) and the previous crop (C for corn, S for soybean) (**Table 1**).

**Table 1:** Planting date, hybrid, seeding rate, previous crop, and tillage practice for the three field trials conducted in 2021.

Field trial	Experiment area (ha)	Planting date	Emergence	Hybrid	Seeding rate (seeds ha^-1^)	Previous crop	Tillage practice	Field trial acronym
Davis-Purdue Agricultural Center	10.6	1-Jun	10-June	Pioneer P1108Q	74,100	Soybean (*Glycine max* L.)	Strip-till	ST-S
Commercial farm (field 1)	13.0	27-Apr	18-May	Dekalb DKC 63-61	83,980	Corn	Conventional	CT-C
Commercial farm (field 2)	25.6	22-May	26-May	Dekalb DKC 62-51	85,5450	Soybean	Conventional	CT-S

Fields were planted using commercial planters. Corn rows were spaced 0.76 m apart and oriented in an east-west direction at CT-C and ST-S, and in a north-south direction at CT-S. Temperature and precipitation data for the 2021 growing season (April to October) were collected from automated weather stations near each site using the Midwestern Regional Climate Center’s cli-MATE online data portal (https://mrcc.purdue.edu/CLIMATE/). Average monthly air temperature and accumulated precipitation information for all trials are presented in **Supplementary Table 2**. There were early planting delays due to unexpected cold and snow in April at all fields. During the crop reproductive phase, favorable moisture conditions were observed at ST-S, while CT-C and CT-S experienced relatively drier conditions.

**Figure 1:**
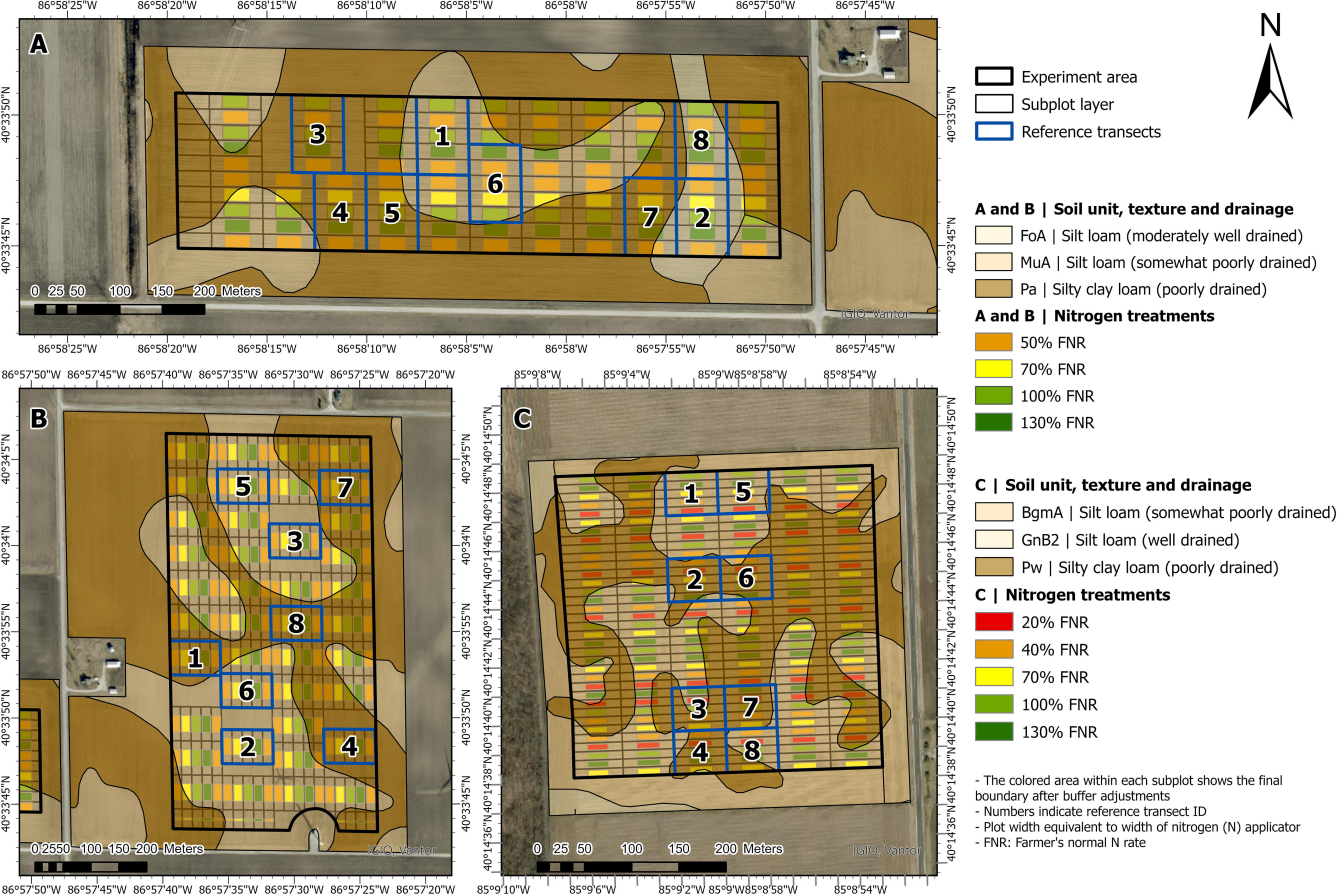
Experiment plot design of three field trials evaluated in this study. Individual letters (A to C) represent each trial (A, CT-C; B, CT-S; C, ST-S). Field names indicate tillage (ST = strip-till; CT = conventional tillage) and previous crop (C, corn; S, soybean). The experimental area within each field is outlined in black. Colored rectangles represent individual subplots receiving different N rates applied at early vegetative growth stages and expressed as a percentage of the farmer’s normal N rate (FNR; A = 222, B = 180, and C = 222 kg N ha^-1^). Reference transects, which include a full replication of all N rates, are outlined in blue. Colored polygons indicate soil series, including associated texture and drainage class (see legend for soil classifications and N treatment levels).

Nitrogen (N) fertilizer rate treatments were applied at each field trial. Four or five N rates and multiple replications were established after planting based on the farmer’s normal N rate (FNR) (**Table 2**; **Figure 1**). Nitrogen rates were applied in plots that were equivalent to the width of the N applicator (9.14 m at ST-S and 18.29 m at CT-C and CT-S) by the length of the field (330 m at ST-S, 305 m at CT-C, and 707 m at CT-S). Information of treatments, N rates, replications, and fertilizer source and timing for each field trial are detailed in **Table 2**. In addition to the N treatments applied after planting, a second application of N was conducted prior to flowering in specific plots only at ST-S. These plots were not considered for this study, as it focused exclusively on areas that received a single N application. Application of other nutrients (phosphorus, potassium, and sulfur) was done uniformly at a single rate across the experimental area in all field trials.

**Table 2:** Farmer’s normal N rate (FNR), treatments, N rates, number of replications, and N fertilizer source and timing for the three field trials conducted in 2021.

Field trial	Farmer’s normal N rate (FNR) (kg ha^-1^)	Treatment ^a^	N rate (kg ha^-1^)	Replications	N source	Treatment application date and stage
ST-S	222	20% FNR^b^	44	7	Urea ammonium nitrate (28% N)	6-Jun (VE)
		40% FNR	89			
		70% FNR	155			
		100% FNR	222			
		130% FNR	289			
CT-C	222	50% FNR^c^	111	2	Anhydrous ammonia (82% N)	5-Jun (V3)
		50% FNR	111			
		70% FNR	155			
		100% FNR	222			
		130% FNR	289			
CT-S	180	50% FNR	90	4	Anhydrous ammonia (82% N)	5-Jun (V2)
		50% FNR	90			
		70% FNR	126			
		100% FNR	180			
		130% FNR	234			

a Lowest %FNR treatment varied depending on the capabilities of the N applicator at each field trial.

b 20%FNR treatment was applied as starter fertilizer at ST-S. No starter fertilizer applied for CT-C and CT-S.

c At CT-C and CT-S, 50%FNR treatment was duplicated for purposes of a separate study.

### 2.2 Subplot delineation and establishment of reference transects

For each field trial, the plot layer was imported into ArcGIS Pro ^©^ 2020 Esri (Esri, Redlands, CA, USA) as a polygonal shapefile. Plots were further delineated into smaller sections (subplots), which were equal to the plot width by 55 m long at ST-S, and 61 m long at CT-C and CT-S. Once the subplots were delineated, a buffer of 1.5 m between adjacent subplots and 15 m between consecutive subplots was applied to avoid potential border effects caused by different N rates. Adjacent subplots representing the range of all N rates were considered as one transect (**Figure 1**). This transect-based configuration follows the conceptual framework of calibration strip or ramped calibration strip approaches used in in-season nitrogen management (Raun et al., 2008), where a gradient of N rates is established within a uniform soil area to evaluate crop responsiveness.

For calculation of the agronomic optimum nitrogen rate (AONR) during the growing season (AONRvi) and the yield-based AONR (AONRy), eight reference transects were established at each of the three field trials. Establishment of reference transects was determined based on the soil types of each field, ensuring that the soil type would be consistent across each transect (**Figure 1**). Spatial soil data was retrieved from the Web Soil Survey (https://websoilsurvey.nrcs.usda.gov/app/). At ST-S, the reference transects did not receive the second application of N to ensure the AONRy could be calculated. At CT-C and CT-S, the reference transects were moved slightly to have a consistent soil type within each transect (**Figure 1**). Transects that overlapped with the reference transects were discarded.

### 2.3 Grain yield measurements

Grain yield data were collected using commercial combines equipped with calibrated, GPS-enabled yield monitors. Data were processed using Ag Leader^®^ SMS™ Advanced (Ag Leader Technology, Ames, Iowa, USA), ArcGIS Pro ^©^ 2020, and RStudio (Posit PBC, Boston, MA, USA) in R version 4.3.0 (R Foundation for Statistical Computing, Vienna, Austria). Yield points outside the study area, with abnormal combine heading, grain moisture (<10% or >33%), or travel speed (<0.7 or >8.2 mph) were removed. Points with speed changes >15% from the previous reading were also excluded to reduce errors from abrupt machine behavior. All remaining yield values were then adjusted to a standard moisture content of 150 g kg^-^¹ (15%), and extreme outliers beyond ±3 standard deviations from the treatment mean were removed.

### 2.4 Satellite imagery acquisition, processing, and data extraction

PlanetScope (www.planet.com) multispectral satellite images (3-m spatial resolution) were retrieved at multiple growth stages between V7 and R6. Selection of the satellite images was based on cloud-free images available for the targeted growth stages. A total of seven growth stages were evaluated. Dates of satellite imagery acquisition are given in **Table 3**. Images were downloaded in GeoTIFF format to preserve geospatial information. The “Surface Reflectance” asset, which includes atmospheric correction, was selected along with the “Harmonize” option to normalize reflectance values to the Sentinel-2 spectral response, enhancing radiometric consistency across dates. Spectral bands (wavelength) Blue (455–515 nm), Green (500–590 nm), Red (590–670 nm), and Near Infrared (780–860 nm) were used for this study.

**Table 3:** Date of acquisition of Planet satellite imagery evaluated, growth stage, timing, days after nitrogen treatment application (DAT), and accumulated precipitation from treatment application for the three field trials conducted in 2021.

Field trial	Date	Growth stage ^a^	Timing ^b^	DAT ^c^	Accumulated precipitation from treatment application (mm)
ST-S	5-Jul	V8	1	29	64
	15-Jul	V10	2	39	116
	19-Jul	V15	3	43	142
	5-Aug	R2	4	60	163
	2-Sep	R4	5	88	201
	10-Sep	R5.25	6	96	235
	26-Sep	R6	7	112	326
CT-C	17-Jun	V8	1	12	15
	22-Jun	V10	2	17	35
	3-Jul	V15	3	28	116
	14-Jul	R1	4	39	167
	30-Jul	R3	5	55	195
	15-Aug	R5	6	71	207
	16-Sep	R6	7	103	250
CT-S	22-Jun	V7	1	17	35
	3-Jul	V11	2	28	116
	14-Jul	V16	3	39	167
	30-Jul	R2	4	55	195
	15-Aug	R4	5	71	207
	31-Aug	R5	6	87	244
	25-Sep	R6	7	112	311

a Vegetative stages were determined based on the leaf collar method (Abendroth et al., 2011).

b Timing: 1 (V7 and V8), 2 (V10 and V11), 3 (V15 and V16), 4 (R1 and R2), 5 (R3 and R4), 6 (R5 and R5.25), and 7 (R6).

c Nitrogen treatment application: ST-S (6-Jun, VE), CT-C (5-Jun, V3), and CT-S (5-Jun, V2).

Satellite imagery were imported into RStudio using the function “brick()” from the “raster” R package. Prior to vegetation index (VI) calculation, pixel values were rescaled to reflectance values ranging from 0 to 1. PlanetScope surface reflectance products are originally stored as scaled integers (0–10000), where reflectance values are multiplied by 10,000 to preserve precision. Therefore, all spectral bands were divided by 10,000 before computing VIs to ensure accurate VI calculation. Sixteen VIs were calculated for each acquisition date, eight RGB-based and eight NIR-based (**Table 4**). The subblock layer of each field trial was used for extracting mean values of the metrics of interest. For the VIs calculated from the satellite imagery, mean values per subblock were extracted in RStudio using the function “extract()” from the “raster” R package.

**Table 4:** Vegetation indices (VIs), their formulas, and the researchers who first developed each VI.

Vegetation index	Index full name	Formula^a^	Reference
RGB-based VI			
EXG	Excess Greenness Index	[2G-R-B]	Woebbecke et al. (1995)
EXR	Excess Red Index	[(1.4R)-B]	Meyer et al. (1999)
VDVI	Visible-band Difference Vegetation Index	[(2G-B-R)/(2G+B+R)]	Wang et al. (2015)
PPRB	Plant Pigment Ratio	[(G-B)/(G+B)]	Metternicht (2003)
VARI	Visible Atmospherically Resistant Index	[(G-R)/(G+R-B)]	Gitelson et al. (2002)
VIG	Vegetation Index Green	[(G-R)/(G+R)]	Tucker (1978)
DVI	Green-Red Difference Vegetation Index	G-R	Tucker (1978)
GREEN	Green spectral band	G	na
NIR-based VI			
NDVI	Normalized Difference Vegetation Index	[(NIR-R)/(NIR+R)]	Rouse et al. (1973)
GNDVI	Green Normalized Difference Vegetation Index	[(NIR-G)/(NIR+G)]	Gitelson et al. (1996)
OSAVI	Optimized Soil-Adjusted Vegetation Index	[(NIR-R)/(NIR+R+0.16)]	Baret et al. (1993)
SAVI	Soil-Adjusted Vegetation Index	(1+L) × [(NIR-R)/(NIR+R+L)], where L = 0.5	Huete (1988)
CVI	Chlorophyll vegetation Index	[(NIR/G) × (R/G)]	Vincini et al. (2008)
RDVI	Renormalized Difference Vegetation Index	[(NIR-R)/√ (NIR+R)]	Roujean and Breon (1995)
GARI	Green Atmospherically Resistant Vegetation Index	NIR-[G-1.7(B-R)]/NIR+[G-1.7(B-R)]	Gitelson et al. (1996)
EVI2	Two-Band Enhanced Vegetation Index	[2.5 × (NIR-Red)]/ [NIR + (2.4 × Red) + 1]	Jiang et al. (2008)

a B, Blue band; G, Green; R, Red; and NIR, Near infrared.

### 2.5 Statistical analysis

**Figure 2:**
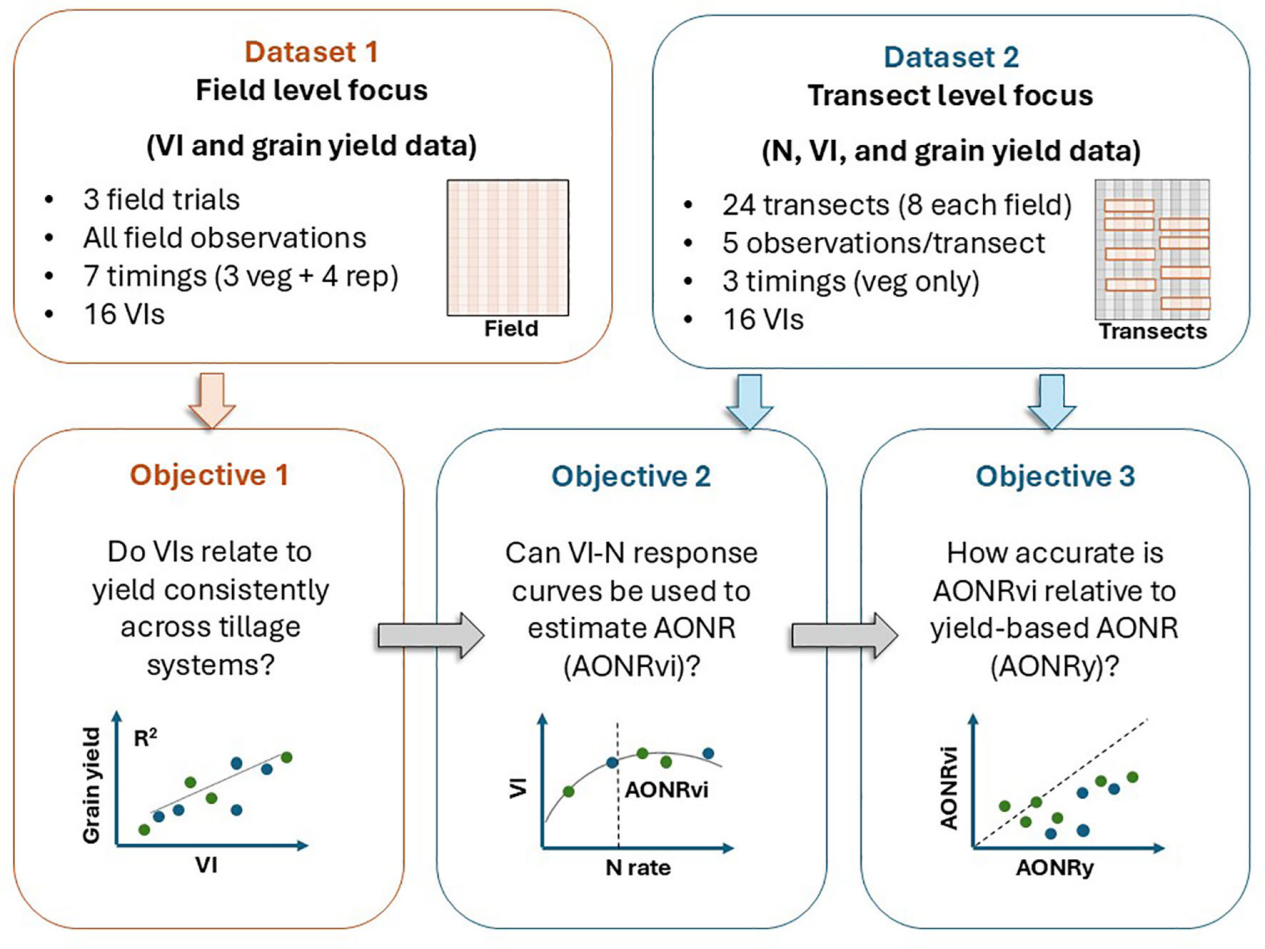
Study workflow showing the datasets used to address the three research objectives.

All statistical analyses were conducted using R version 4.3.0 through the RStudio interface. **Figure 2** shows an overview of the dataset used for each objective. Field-level VI and yield data (Dataset 1) were used to assess VI–yield relationships across field trials under different tillage systems and crop residue conditions (Objective 1). Transect-level N rate, VI, and grain yield data (Dataset 2) were used to fit VI–N and yield–N response curves to estimate AONR rates derived from VI (AONRvi) and yield (AONRy) (Objective 2), and to assess agreement between AONRvi and AONRy (Objective 3).

#### 2.5.1 Objective 1

To evaluate the strength of relationship between satellite-derived VIs and grain yield, simple linear regressions were fitted between grain yield (dependent variable) and each VI (independent variable). Regressions were performed for each combination of field, timing, and VI, resulting in a total of 336 models (3 fields × 7 timings × 16 VIs). For each model, the coefficient of determination (R²) (Equation 1) and the normalized root mean square error (nRMSE; RMSE divided by the range of observed yield values) (Equations 2, 3) were calculated to assess model fit and predictive performance. The normalized RMSE was used instead of absolute RMSE to enable comparisons across sites with different grain yield ranges. Models with p < 0.10 were considered statistically significant.

(1) $${\text{R}}^{2}=1-\frac{\sum_{i=1}^{n}{\left(x_{i}-y_{i}\right)}^{2}}{\sum_{i=1}^{n}{\left(x_{i}-\bar{x}\right)}^{2}}$$

(2) $$\text{RMSE}=\sqrt{\frac{1}{n}\sum_{i=1}^{n}{\left(y_{i}-{\hat{\text{y}}}_{i}\right)}^{2}}$$

(3) $$\text{nRMSE}=\frac{RMSE}{y_{max}-y_{min}}$$

#### 2.5.2 Objective 2

To identify site-specific responses to N, each transect within a field was treated as an individual experimental unit (n = 24; 8 transects per field trial) (**Figure 1**). For each transect, 16 VIs (**Table 4**) and three timings were evaluated (**Table 3**, timings 1 to 3), resulting in 1152 individual VI–N response curves (24 transects × 16 VIs × 3 timings), each with five observations. Both quadratic-plateau and linear-plateau models were fitted to describe the response of each VI to applied N rates, as well as to the yield-based N response for comparison. Timings corresponding to the vegetative period were selected because they align with practical in-season N decision windows.

Only models with coefficients of determination (R²) > 0.50 were retained, as lower R² values are typically associated with small or inconsistent yield response to N and high variability rather than true N treatments effects (Scharf et al., 2005). Then, retained models were screened for agronomic plausibility, accepting only linear-plateau models with positive slopes and quadratic-plateau models with concave-down responses. These criteria ensured that the models reflected the expected biological saturation of the crop, where vegetation indices increase with nitrogen supply until reaching a physiological limit. When both model types met these criteria for a given VI-N or yield-VI response curve, the model with lower Akaike Information Criterion (AIC) (Akaike, 1974) was selected. If both had the same AIC, the quadratic-plateau model was preferred (Cerrato and Blackmer, 1990). The AONR estimated from VI-based models (AONRvi) and that derived from yield response (AONRy) were extracted from the selected model for each transect. For VI-N curves in which AONRvi exceeded the maximum N rate tested within a given field trial, the VI-N curve was discarded.

#### 2.5.3 Objective 3

The percent difference between AONRvi and AONRy (Equation 4) was calculated to assess accuracy of AONRvi. The agreement between observed (AONRy) and estimated (AONRvi) values was also evaluated using the coefficient of determination derived from the Pearson correlation between the two variables (R^2^ = r^2^) (Equation 5). Model accuracy was further quantified using the root mean square error (RMSE) and mean absolute error (MAE) (Equation 6).

(4) $$\%\text{ AONRvi}-\text{AONRy}=(\frac{AONRvi-AONRy}{AONRy})*100$$

(5) $$\text{r}=\frac{n(\sum xy)-(\sum x)(\sum y)}{\sqrt{\left[n\sum x^{2}-{\left(\sum x\right)}^{2}][n\sum y^{2}-{\left(\sum y\right)}^{2}\right]}}$$

(6) $$\text{MAE}=\frac{1}{n}\sum_{i=1}^{n}\left|y_{i}-{\hat{\text{y}}}_{i}\right|$$

## 3 Results

### 3.1 Relationship between vegetation indices and corn grain yield under different tillage systems and crop residue scenarios

At the field level, a total of 336 regression models were evaluated (3 field trials x 7 timings x 16 VI), 144 corresponding to the vegetative period (3 field trials x 3 timings x 16 VIs) and 192 to the reproductive period (3 field trials x 4 timings x 16 VI). Of these, 85 (59%) and 170 (89%) were significant (*p* < 0.10), respectively (**Table 5**).

**Table 5:** Number of significant regression models (p < 0.10) and range of coefficients of determination (R²) and normalized RMSE (nRMSE) for relationships between vegetation indices (VIs) and grain yield across growth periods, VI type, and field trials. Only significant models were included in the summary.

Period	VI type ^a^	Field ^b^	Models	Significant models	min R^2^	max R^2^	min nRMSE	max nRMSE
Veg	ALL	ALL	144	85	0.02	0.29	0.15	0.24
	NIR	ALL	72	55	0.03	0.29	0.15	0.24
		CT-C	24	24	0.11	0.29	0.15	0.17
		CT-S	24	19	0.03	0.26	0.18	0.20
		ST-S	24	12	0.07	0.25	0.22	0.24
	RGB	ALL	72	30	0.02	0.24	0.16	0.24
		CT-C	24	17	0.03	0.24	0.16	0.18
		CT-S	24	6	0.02	0.06	0.20	0.20
		ST-S	24	7	0.07	0.24	0.22	0.24
Rep	ALL	ALL	192	170	0.01	0.84	0.07	0.24
	NIR	ALL	96	88	0.13	0.84	0.07	0.22
		CT-C	32	30	0.13	0.84	0.07	0.17
		CT-S	32	26	0.17	0.58	0.13	0.19
		ST-S	32	32	0.25	0.81	0.11	0.22
	RGB	ALL	96	82	0.01	0.81	0.08	0.24
		CT-C	32	29	0.04	0.80	0.08	0.18
		CT-S	32	31	0.01	0.61	0.13	0.20
		ST-S	32	22	0.13	0.81	0.11	0.24

a Sixteen vegetation indices (VIs) were evaluated (eight per VI type). Names and formulas are provided in **Table 4**.

b Field names indicate tillage (ST, strip-till; CT, conventional tillage) and previous crop (C, corn; S, soybean).

During the vegetative period, coefficients of determination (R^2^) values ranged from 0.02 to 0.29, with NIR-based VIs showing stronger relationships with grain yield than RGB-based VIs. Of the 72 models evaluated for each spectral group, 55 NIR-based models were significant compared with 30 RGB-based, with R² values ranging from 0.03 to 0.29 for NIR and 0.02 to 0.24 for RGB (**Table 5**). At the field-trial level, although the total number of models evaluated was the same for each site (n = 48; 24 NIR-based and 24 RGB-based), the number of significant models differed among trials. The conventionally tilled corn–corn field (CT-C) had the highest number of significant models (n = 41; 24 NIR-based and 17 RGB-based), followed by the conventionally tilled soybean–corn field (CT-S) (n = 25; 19 NIR-based and 6 RGB-based). The strip-till soybean–corn field (ST-S) consistently showed the fewest significant relationships (n=19; 12 NIR-based and 7 RGB-based).

**Figure 3:**
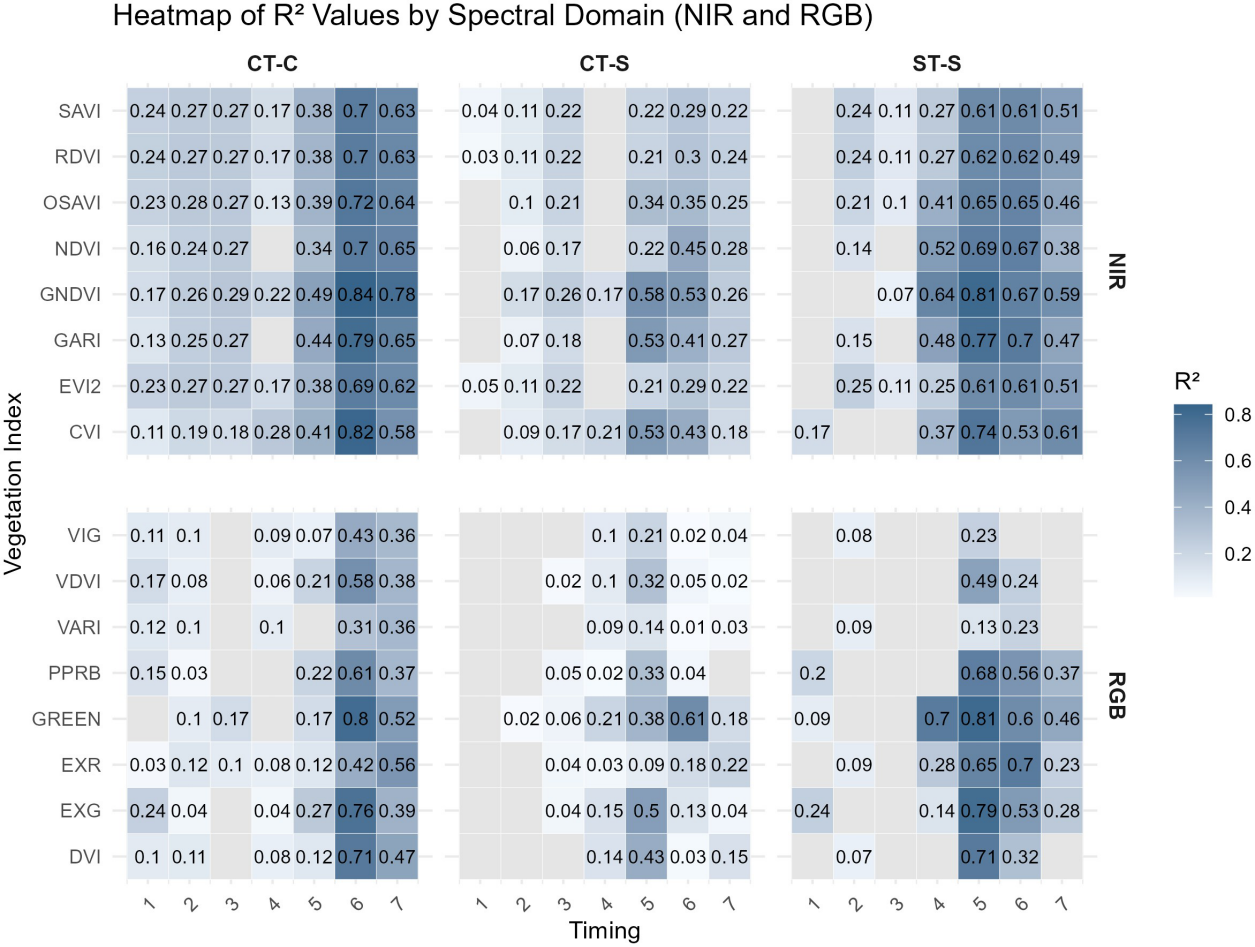
Heatmap of coefficients of determination (R²) between vegetation indices (VIs) and grain yield across three field trials (CT-C, CT-S, ST-S) and timings (1, V7 and V8; 2, V10 and V11; 3, V15 and V16; 4, R1 and R2; 5, R3 and R4; 6, R5 and R5.25; and 7, R6). Gray cells indicate the regression model was not significant (p≥0.10). VIs names and formulas are described in **Table 4**.

The number and strength of significant VI–yield relationships increased from the vegetative to the reproductive period (**Figure 3**). Across all field trials, R² values increased from early to late growth stages, reaching their highest values during reproductive stages. Corresponding ranges of R^2^ and normalized RMSE values by field trial and timing are provided in **Supplementary Table 3**.

### 3.2 Feasibility of estimating AONR using VI-N response curves

At the transect level, a total of 1152 VI–N response curves were evaluated (24 transects × 3 timings × 16 VIs). Of these, 192 curves (17%) met the selection criteria and were retained for estimating AONRvi (**Table 6**). At the field-trial level, each site contributed 384 VI–N response curves (8 transects × 3 timings × 16 VIs). The proportion that remained after applying the selection criteria ranged from 14 to 20% across fields. The CT-S field trial resulted in the highest proportion of usable curves (20%; n = 75), followed by CT-C (16%; n = 62), and ST-S (14%; n = 55) (**Table 6**).

**Table 6:** Total number of vegetation index (VI)–N rate response curves evaluated by field trial and timing, and corresponding percentage of curves that met the criteria for estimation of VI-based agronomic optimum nitrogen rate (AONRvi).

Field ^a^	Timing ^b^	Total N-VI curves	Usable curves (n)	Usable curves (%)
All	All	1152	192	16.7
CT-C	All	384	62	16.1
	1	128	22	17.2
	2	128	18	14.1
	3	128	22	17.2
CT-S	All	384	75	19.5
	1	128	35	27.3
	2	128	25	19.5
	3	128	15	11.7
ST-S	All	384	55	14.3
	1	128	7	5.5
	2	128	29	22.7
	3	128	19	14.8

a Field names indicate tillage (ST, strip-till; CT, conventional tillage) and previous crop (C, corn; S, soybean).

b Timing, 1 (V7 and V8), 2 (V10 and V11), 3 (V15 and V16).

**Figure 4:**
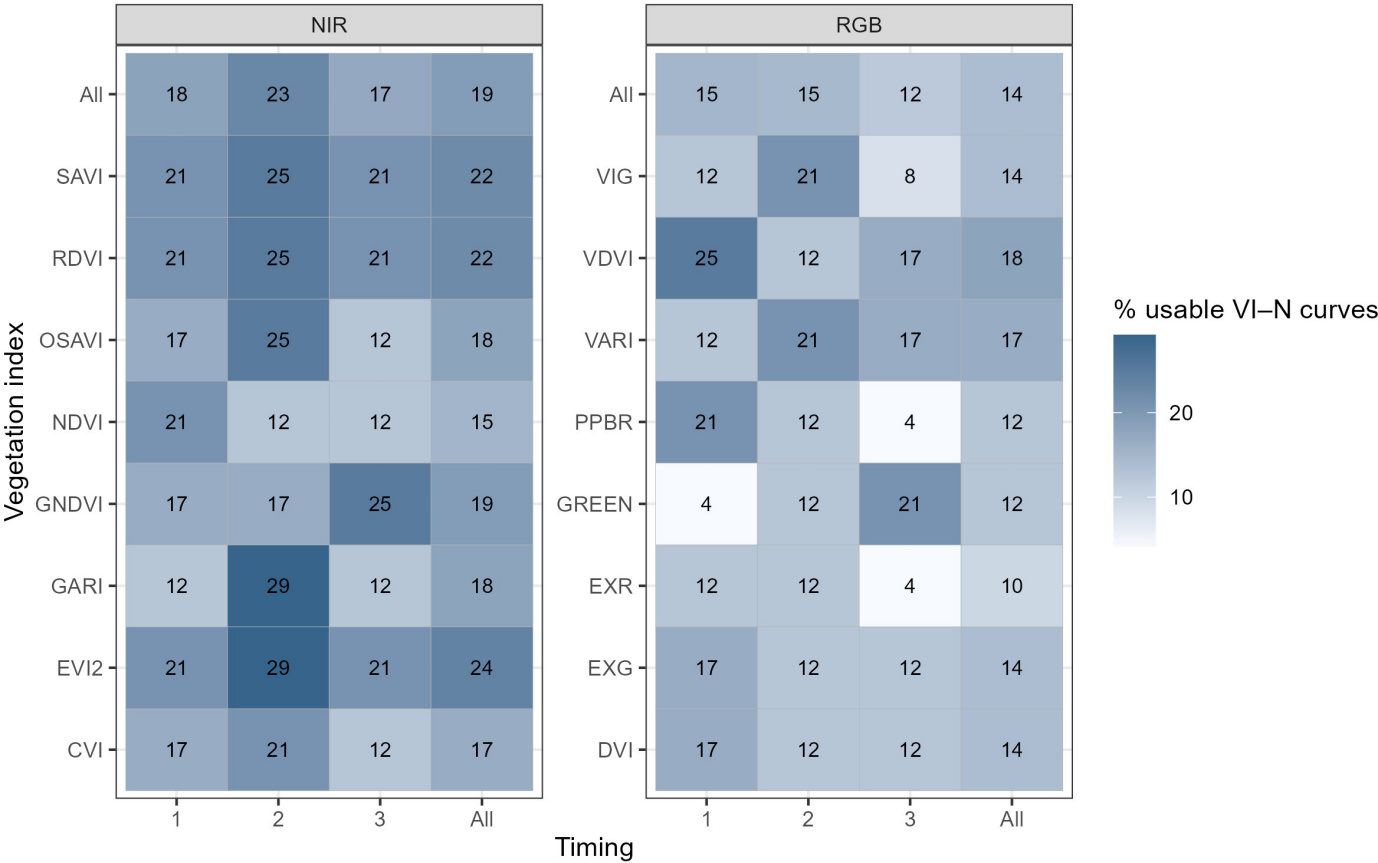
Percentage of VI–N response curves retained after applying model selection criteria. Values are shown by vegetation index (rows) and image timing (columns; 1, V7 and V8; 2, V10 and V11; 3, V15 and V16), including overall counts across timings and VIs (“All”). Panels are separated by VI type: near-infrared (NIR)-based indices (left) and RGB-based indices (right). VIs names and formulas are described in **Table 4**.

Each VI was evaluated in 72 VI–N curves (24 transects × 3 timings). Across all fields and timings, the proportion of VI-N curves retained for estimating AONRvi ranged from 10 to 24% depending on the specific VI (**Figure 4**). NIR-based indices consistently outperformed RGB-based indices, with retained proportions of 15–24% compared to 10–18%, respectively. Within the NIR group, EVI2 showed the highest proportion (24%), and VDVI among the RGB-based indices (18%) (**Figure 4**).

Image acquisition timing influenced the proportion of VI–N curves retained for AONRvi across field trials. For NIR-based indices, the highest proportion was at timing 2 (23%), followed by timing 1 (18%) and timing 3 (17%). RGB-based indices showed lower and less variable retention across timings (12–15%), with the lowest values observed at Timing 3 (12%) (**Figure 4**).

**Figure 5:**
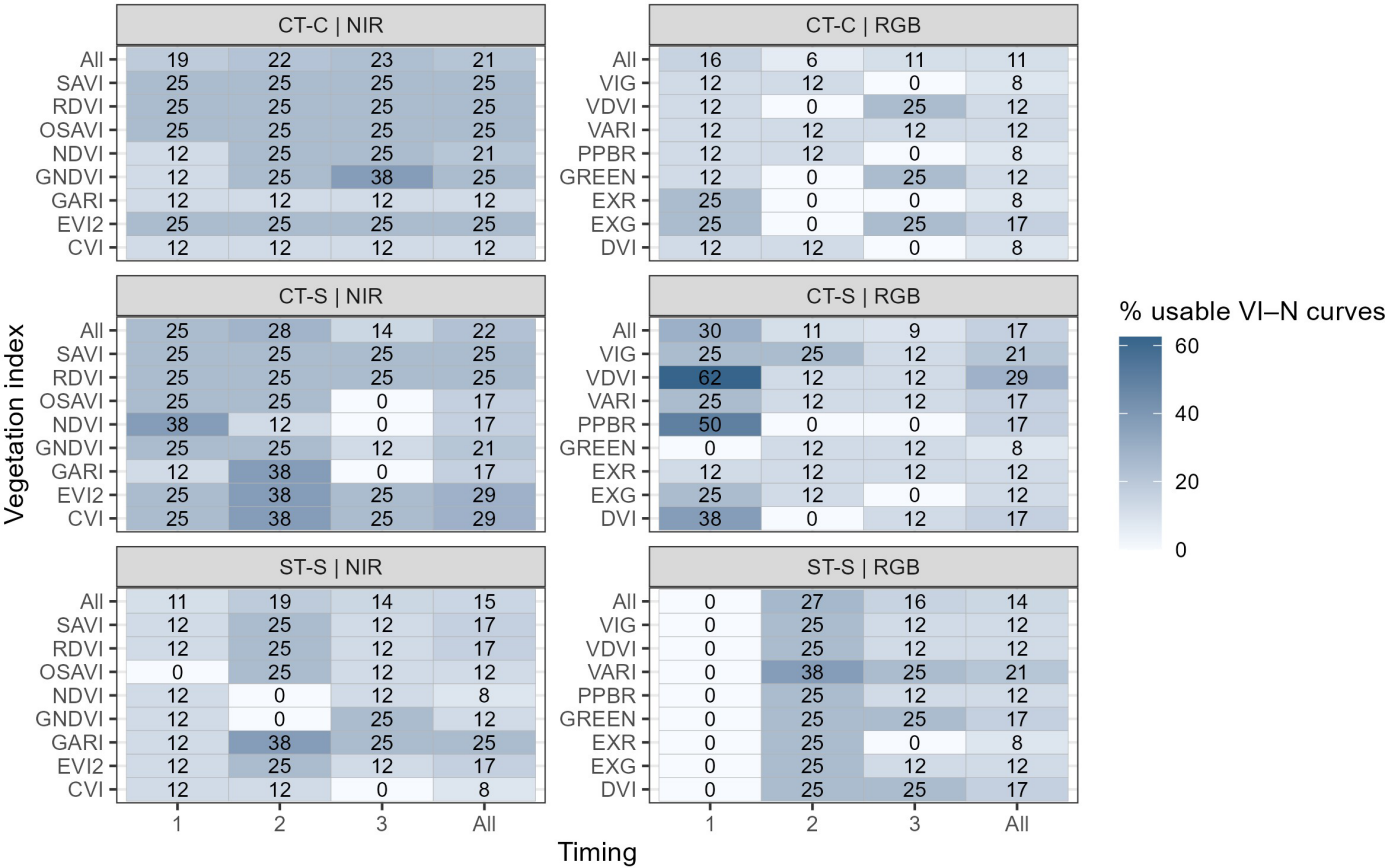
Percentage of VI–N response curves retained after applying model selection criteria, separated by field trial. Field names indicate tillage (ST, strip-till; CT, conventional tillage) and previous crop (C, corn; S, soybean). Values are shown by vegetation index (rows) and image timing (columns; 1, V7 and V8; 2, V10 and V11; 3, V15 and V16), including overall counts across timings and VIs (“All”). Panels are separated by VI type: near-infrared (NIR)-based indices (left) and RGB-based indices (right). VIs names and formulas are described in **Table 4**.

Differences in VI performance were observed among field trials (**Figure 5**). The CT-S field trial showed higher proportions of usable VI-N curves across multiple VIs and growth stages. In contrast, ST-S showed lower proportions overall, with several VI–timing combinations yielding few or no curves that met the criteria. The conventionally tilled corn–corn field (CT-C) showed intermediate patterns, with variability depending on vegetation index and image timing.

### 3.3 Accuracy of VI-based (AONRvi) compared to yield-based AONR (AONRy)

**Figure 6:**
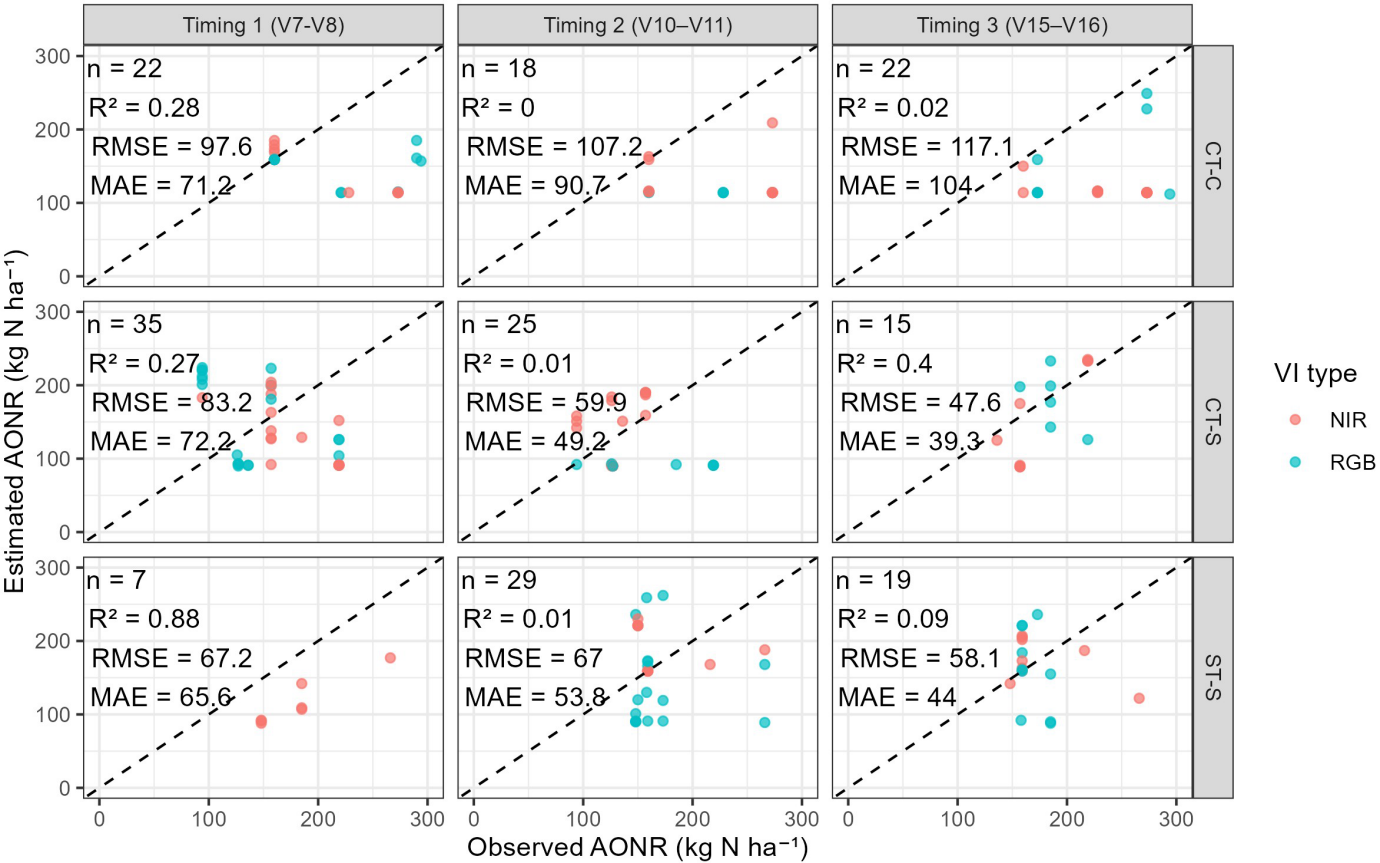
Observed (AONRy) vs. estimated agronomic optimum N rate from vegetative indices (AONRvi) for all retained VI–N response models that met the selection criteria, separated by field trial and timing. Dashed line represents the 1:1 relationship between AONRvi and AONRy. Field names indicate tillage (ST, strip-till; CT, conventional tillage) and previous crop (C, corn; S, soybean). VIs names and formulas are described in **Table 4**.

**Figure 6** illustrates the relationship between observed (AONRy) and estimated AONR (AONRvi) across field trials and timings. There are points distributed both above and below the 1:1 line, indicating instances of over- and underestimation. Agreement between AONRy and AONRvi varied widely among field trials and timings, and coefficients of determination were generally low, reflecting the scatter in the relationship.

**Figure 7:**
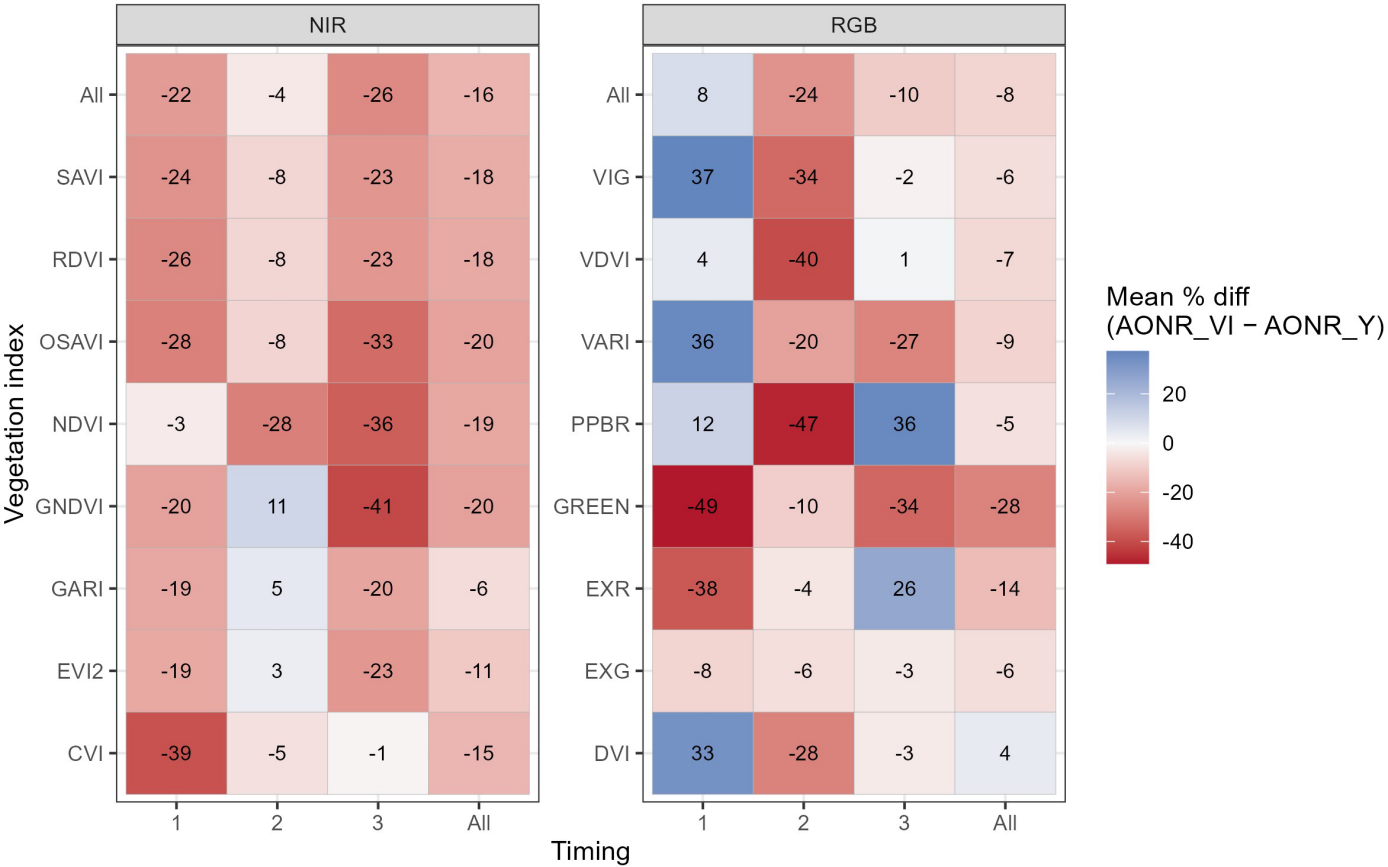
Percent differences between estimated (AONRvi) and observed AONR (AONRy). Values are shown by vegetation index (rows) and image timing (columns; 1, V7 and V8; 2, V10 and V11; 3, V15 and V16), including overall counts across timings and VIs (“All”). Panels are separated by VI type: near-infrared (NIR)-based indices (left) and RGB-based indices (right). Field names indicate tillage (ST, strip-till; CT, conventional tillage) and previous crop (C, corn; S, soybean). VIs names and formulas are described in **Table 4**.

Across all VIs and field trials, mean percent differences between estimated and observed AONR were predominantly negative, indicating a tendency toward underestimation (**Figure 7**). When averaged across all VIs, mean differences ranged from −26 to 8% depending on timing. For NIR-based indices, mean differences ranged from −26 to −4%, and from −24 to 8% for RGB. While no consistent trend with timing was evident, Timing 2 exhibited the smallest overall deviation from AONRy (−4%) for NIR-based indices (**Figure 7**).

**Figure 8:**
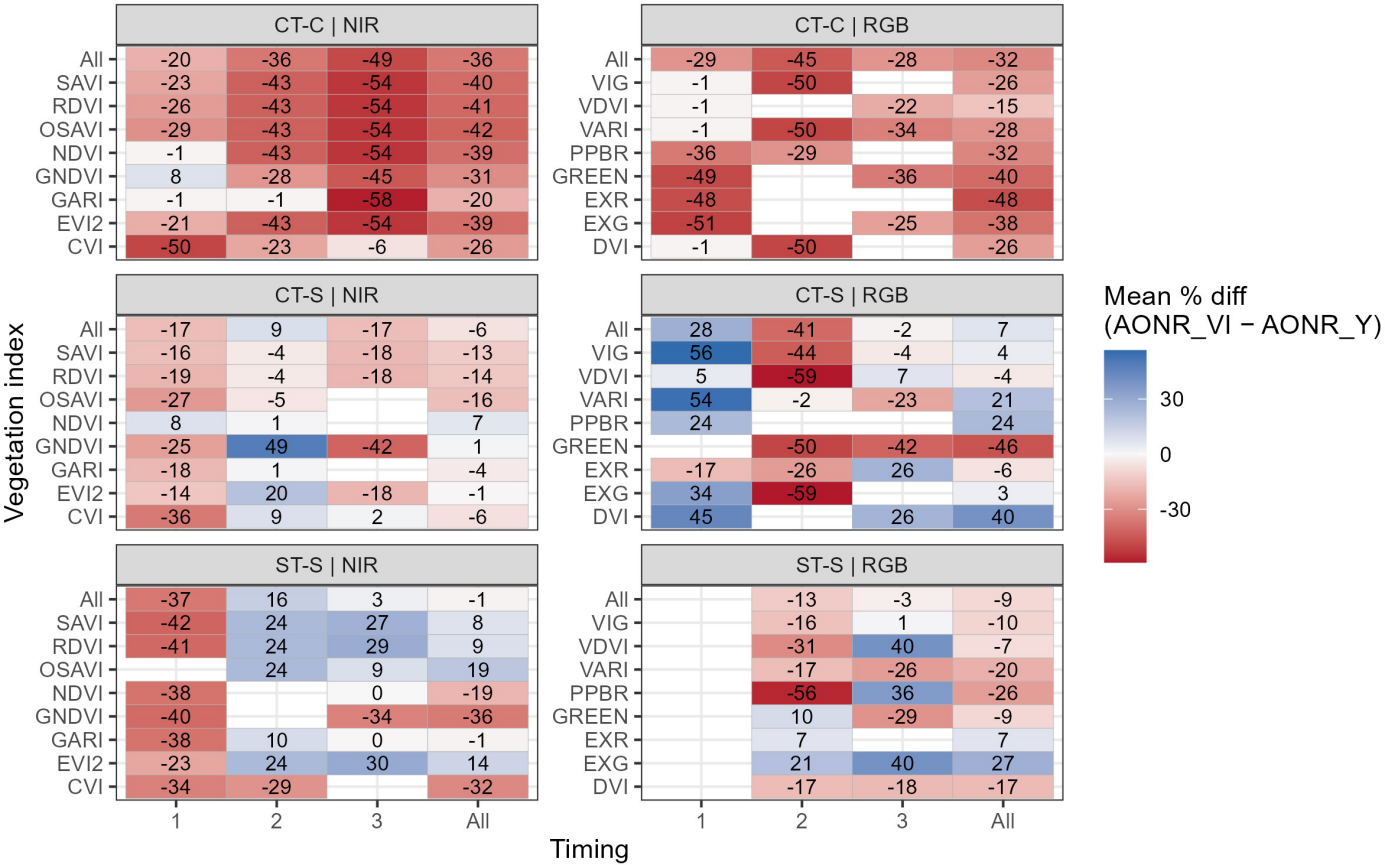
Percent differences between estimated (AONRvi) and observed AONR (AONRy), separated by field trial. Values are shown by vegetation index (rows) and image timing (columns; 1, V7 and V8; 2, V10 and V11; 3, V15 and V16), including overall counts across timings and VIs (“All”). Panels are separated by VI type: near-infrared (NIR)-based indices (left) and RGB-based indices (right). Field names indicate tillage (ST, strip-till; CT, conventional tillage) and previous crop (C, corn; S, soybean). VIs names and formulas are described in **Table 4**.

There were differences in AONR accuracy across field trials (**Figure 8**). In the CT-C trial, most VI–timing combinations resulted in negative mean differences, with values ranging from −58 to 8%. In contrast, the conventionally tilled soybean–corn (CT-S) and strip-till soybean–corn (ST-S) fields showed a wider spread, including both under- and overestimation, with mean differences ranging from −59 to 56% depending on VI and timing.

Differences among VIs were also evident. Most NIR-based indices showed negative mean differences across timings, indicating consistent underestimation relative to AONRy. RGB-based indices exhibited greater variability, including several cases of positive mean differences, with values reaching up to 56%. Although AONRvi tended to underestimate AONRy on average, field-level results in this study showed variability across VIs and timings, indicating that VI performance was field- and timing-dependent rather than universally consistent (**Figure 8**).

## 4 Discussion

This study evaluated the potential of 3-m satellite-derived VIs to predict corn yield and estimate AONR across multiple growth stages and environments. While VIs demonstrated moderate ability to explain yield variability and to generate biologically meaningful N response curves, their predictive accuracy and consistency varied among growth stages, VIs, and field management conditions.

### 4.1 Strength of relationships between satellite-derived vegetation indices and corn grain yield

Across all field trials, the strength of relationships between VIs derived from satellite imagery and grain yield increased from the vegetative to the reproductive period (**Figure 3**). During the vegetative period, maximum coefficients of determination (R²) were lower (≤ 0.31) compared to the reproductive stages (R² up to 0.84). This pattern is well documented in the literature (Barzin et al., 2020; Killeen et al., 2024; Kumar et al., 2023; Oglesby et al., 2022; Sunoj et al., 2021) and reflects the increasing influence of late-season physiological processes, such as pollination, kernel set, and grain filling on final yield (Alqudah et al., 2011; Bennett et al., 1989). As recently reviewed by Xiao et al. (2025), while remote sensing accurately captures these processes at regional scales, field-scale yield forecasting remains challenging due to the complex interaction of canopy structure, biochemical parameters, and localized environmental stressors.

Field-to-field differences in early-season VI–yield relationships illustrated the influence of soil background and crop residue on spectral response, supporting hypothesis H1. The strip-till trial (ST-S), characterized by higher crop residue, showed fewer significant models than the conventionally tilled fields (CT-C and CT-S) (**Table 5**, **Figure 3**). During early vegetative stages, reduced canopy cover likely increased the contribution of soil and crop residue reflectance within each pixel, particularly at the 3-m spatial resolution used in this study (Gausman et al., 1975; Psomiadis et al., 2016; Rocchini, 2007). Similar limitations have been reported for satellite, UAV, and proximal sensing platforms, where soil and residue interference reduced the sensitivity of VIs to crop status early in the season (Scharf and Lory, 2002; Bausch and Khosla, 2010; Kumar et al., 2023; Rambo et al., 2010; Jones et al., 2015).

Beyond background effects, weaker VI–yield relationships during the vegetative period compared to the reproductive period reflect the limited ability of early-season canopy variability to capture processes that ultimately determine final yield. Consistent with hypothesis H2, differences among fields were less pronounced during reproductive stages, when near-complete canopy closure likely reduced soil and residue interference (**Figure 3**).

Overall, vegetative-stage VI–yield relationships were constrained by canopy development and background effects, particularly under higher crop residue conditions. Although reproductive-stage VIs showed stronger relationships with yield, in-season nitrogen management decisions occur during the vegetative period. Therefore, the following sections focus exclusively on vegetative-stage imagery for evaluating VI–N response and AONR estimation.

### 4.2 Estimation and accuracy of VI-based agronomic optimum N rate (AONRvi)

Only 17% (192 of 1152) of the VI–N response models met the selection criteria for deriving AONRvi, highlighting both the challenge and potential of using satellite-derived VIs for in-season N management (**Table 6**). When a distinct VI response to N rates was detected, it indicated measurable N limitation, suggesting that VIs from satellite imagery (3-m resolution) could serve as a decision tool for sidedress intervention. Conversely, the absence of a detectable response likely reflected conditions where N was not limiting at that time or mild N deficiencies that remain spectrally undetectable during early vegetative stages (Burns et al., 2022). The occurrence of a measurable crop response to N may vary depending on factors such as soil N supply, management practices, and weather conditions (Puntel et al., 2019).

Differences among field trials were consistent with the potential influence of crop residue. The strip-till soybean–corn field (ST-S) produced fewer usable curves than the conventionally tilled fields, whereas the conventionally tilled soybean–corn field (CT-S) showed the highest proportion. These patterns support hypothesis H3, suggesting that greater crop residue in strip-till systems may weaken the spectral response of VI to applied N during the vegetative period.

Image acquisition timing influenced VI–N response detection, with mid-vegetative stages (timing 2, V10-V11) resulting in higher proportions of usable curves (**Figure 4**). Earlier (timing 1, V7–V8), growth stage and soil background likely reduced sensitivity to N rate differences. Also, as reported by Paiao et al. (2020); Burns et al. (2022), the poor predictive performance of early-stage measurements reflects a physiological limitation, as crop N demand is still low and plant growth often occurs under sufficient soil N supply. On the other hand, at later stages (timing 3, V15–V16), spectral saturation due to greater canopy cover potentially diminished the ability to distinguish among N treatments using VIs (Gitelson et al., 1996; Huete et al., 1997). For in-season N management decisions, mid-vegetative stages could provide the best balance between canopy closure and sufficient remaining time for late sidedress N applications (Fernandez et al., 2020). While late-season decisions typically account for various agronomic indicators and management factors, we used AONRvi as a streamlined in-season proxy for yield-based requirements. Although the 17% model success rate poses a scalability challenge, reporting these results is essential for defining the boundary conditions of satellite-based diagnostics.

Agreement between estimated (AONRvi) and observed (AONRy) was variable across field trials, VIs, and image acquisition timings, with a tendency for underestimation (**Figure 7**). This results align with the study by Paiao et al. (2020), where optical sensors (SPAD, GreenSeeker, and RapidSCAN) underestimated AONR at V8, suggesting that canopy reflectance at a single time point cannot fully capture the dynamic post-sensing N uptake and remobilization processes. This reflects the complexity and temporal disconnect between early-season spectral signals and end-of-season N demand, and the limitation of relying solely on vegetative-stage spectral information to infer full-season AONR. While AONRvi is derived from single-time-point canopy reflectance during the vegetative period, AONRy reflects integrated crop responses over the entire growing season.

Among the evaluated timings, images acquired at mid-vegetative stages (V10-V11) generally resulted in smaller mean deviations from AONRy for NIR-based indices compared to earlier or later timings (**Figure 7**). RGB-based indices showed greater variability across stages. This partial support for hypothesis H4 suggests that canopy development at mid-vegetative stages may reduce soil and residue interference while still preserving sensitivity to N rate differences.

Differences in AONRvi accuracy among each VI evaluated highlight the effects of VI selection. NIR-based indices generally exhibited more consistent underestimation, whereas RGB-based indices showed greater variability and more frequent overestimation in some field–timing combinations. The advantage of NIR is its sensitivity to changes in internal leaf structure and chlorophyll content, which are closely linked to plant N status (Huete, 1988). Previous studies using high resolution RGB imagery found stronger correlations of RGB-based VIs with grain yield than NDVI, particularly under controlled plot-level conditions (Vergara-Díaz et al., 2016). However, at the coarser (3 m) satellite scale of this study, RGB-based VIs were likely more affected by soil and residue reflectance, resulting in weaker performance.

At the individual VI level, no single VI consistently provided the most accurate AONRvi estimates across all fields and growth stages. Although GARI showed the smallest mean deviation from AONRy when averaged across fields and timings (−6%) (**Figure 7**), its performance varied by field and timing, with instances of both over- and underestimation (**Figure 8**). GARI exhibited its closest agreement with AONRy at Timing 2 across all field trials. These results indicate that while timing can improve the reliability of VI-based AONR estimates, VI performance remained context dependent and no single index was universally optimal across all the timings and crop management conditions evaluated.

### 4.3 Broader implications and next steps

The results from this study suggest that while estimating AONR from satellite-derived VIs remains challenging, certain combinations of timing and VI type, particularly NIR-based VIs acquired around V10–V11, show potential for operational in-season N management. As satellite spectral and temporal resolution continue to improve, capturing these responsive growth windows may enable more reliable, field-scale prediction of site-specific optimum N rates. The shift toward 3-m resolution commercial imagery mirrors broader trends in digital image processing, where high-resolution optical data are increasingly utilized to bridge the gap between traditional field sampling and automated, large-scale vegetation monitoring (Zheng et al., 2025).

This work advances previous studies by evaluating multiple VI, growth stages, and contrasting crop residue conditions at the field scale to identify factors limiting early-season VI performance. Our results revealed how both spectral domain (NIR vs. RGB) and crop residue can influence the ability of satellite imagery to capture yield and N response, highlighting the importance of context-specific calibration.

These results also highlight an important consideration for broader application. Recent remote sensing studies have shown that models developed under one set of environmental or management conditions do not always perform well when applied to different regions or seasons (Zheng et al., 2024; Li et al., 2025). Although those studies focus on image classification tasks, they emphasize the challenge that variability in surface conditions and imaging context can limit model transferability. In agronomic applications, this suggests that satellite-based N management tools should be validated under local management and environmental conditions rather than assumed to perform consistently across fields or years.

While this study demonstrates the potential and limitations of satellite-derived VIs for estimating in-season AONR, some design considerations should be noted. Analyses intentionally relied on satellite imagery alone to evaluate approaches that are operationally feasible for producers, rather than sensor-intensive methods with limited scalability. Transects were selected to maintain relatively uniform soil conditions across their extent, minimizing within-transect variability unrelated to N rate and allowing clearer interpretation of VI–N responses. This constraint reduced the number of transects per field but prioritized control over confounding soil effects. Additionally, vegetative-stage imagery was the main focus of the study because it aligns with in-season decision windows, even though yield prediction is stronger at later stages. Finally, because imagery was collected prior to Planet’s red-edge band availability, the performance of red-edge-based VIs could not be evaluated.

Future efforts should focus on improving in-season AONR estimation by integrating satellite imagery with complementary, readily available data layers, such as elevation, soil properties, weather, and historical yield, rather than relying mostly on additional sensing platforms. Thereby maintaining both agronomic relevance and practical adoption. Additionally, because the typical sidedress window occurs earlier (V4–V8), a key challenge remains in translating canopy-based diagnostics into timely management actions (Nigon et al., 2020).

## 5 Conclusions

The results from this study showed that 3-m satellite-derived VIs had potential and limitations as indicators of grain yield and for capturing corn response to N. During the vegetative period, VI–yield relationships were generally weak (R² ≤ 0.31) and varied across field trials, with fewer significant relationships observed in the strip-till field compared to conventionally tilled fields. Differences among fields diminished during reproductive stages (R² up to 0.84), illustrating the limitations of early-season VIs as proxies for final yield and reinforcing that vegetative-stage signals capture only a partial expression of yield potential.

When using VIs as proxies for grain yield during the vegetative period to estimate AONRvi, only 17% of the VI–N response curves evaluated across field trials and timings met the selection criteria, with the lowest proportion occurring in the strip-till system. Among the usable VI–N curves, AONRvi estimates showed considerable variability relative to AONRy, including both over- and underestimation. Mid-vegetative imagery (V10–V11) resulted in the smallest deviations from AONRy across field trials. Although GARI showed the smallest overall mean deviation, no single VI consistently outperformed others across all fields and timings. In this study, 3-m satellite imagery was able to detect crop N response under specific conditions. However, its reliability for estimating in-season AONR was strongly influenced by field management, image timing, and spectral domain.

## Data Availability

The datasets generated and analyzed during this study are included in the article and **Supplementary Material**. Additional data are available from the corresponding author upon reasonable request.
