# Supplementary material for: Field-scale evaluation of satellite-derived vegetation indices and image timing for in-season nitrogen management in corn
Source: Front Plant Sci. 2026 Mar 20;17:1731400. doi: 10.3389/fpls.2026.1731400 (PMC13047098; doi:10.3389/fpls.2026.1731400)
Supplement: Supplementary file 1 [file Table1.docx]

Supplementary Material

Table S1. Soil classification and percent of field area by soil type for the three field trials evaluated in the study. Data obtained from: Web Soil Survey.

| Field trial | Field trial acronym | Experiment area (ha) | Slope (%) | Soil series | Taxonomic class |
| --- | --- | --- | --- | --- | --- |
| Davis-Purdue Agricultural Center | ST-S | 10.6 | 0-1 | Pewamo (Pw) | Fine, mixed, active, mesic Typic Argiaquolls |
|  |  |  | 0-2 | Blount (BgmA) | Fine, illitic, mesic Aeric Epiaqualfs |
| Commercial farm (field 1) | CT-C | 13.0 | 0 | Pella  (Pa) | Fine-silty, mixed, superactive, mesic Typic Endoaquolls |
|  |  |  | 0-2 | Mundelein (MuA) | Fine-silty, mixed, superactive, mesic Aquic Argiudolls |
|  |  |  | 0-2 | Foresman (FoA) | Fine-loamy, mixed, active, mesic Oxyaquic Argiudolls |
| Commercial farm (field 2) | CT-S | 25.6 | 0-2 | Mundelein (MuA) | Fine-silty, mixed, superactive, mesic Aquic Argiudolls |
|  |  |  | 0 | Pella  (Pa) | Fine-silty, mixed, superactive, mesic Typic Endoaquolls |
