# Supplementary material for: Field-scale evaluation of satellite-derived vegetation indices and image timing for in-season nitrogen management in corn
Source: Front Plant Sci. 2026 Mar 20;17:1731400. doi: 10.3389/fpls.2026.1731400 (PMC13047098; doi:10.3389/fpls.2026.1731400)
Supplement: Supplementary file 2 [file Table2.docx]

Supplementary Material

**Table S2.** Average monthly air temperature and accumulated precipitation and snow from April 1^st^ to October 31^st^, and deviation from the 30-yr average (1991-2020).

| Month | Air temperature (°C) | | |  | Precipitation (mm) | | |  | Snow (mm) |
| --- | --- | --- | --- | --- | --- | --- | --- | --- | --- |
|  | 20-year monthly average | Monthly average | Deviation from normal |  | 20-year  average precipitation accumulation | Monthly precipitation accumulation | Deviation from normal |  | Monthly snow accumulation |
| ST-S |  |  |  |  |  |  |  |  |  |
| Apr | 10.3 | 10.2 | -0.1 |  | 103 | 59 | -43.4 |  | 76 |
| May | 16.5 | 14.2 | -2.3 |  | 112 | 111 | -0.3 |  | 0 |
| Jun | 21.6 | 22.2 | 0.6 |  | 118 | 67 | -50.5 |  | 0 |
| Jul | 23.1 | 22.7 | -0.4 |  | 112 | 119 | 7.6 |  | 0 |
| Aug | 22.0 | 23.5 | 1.5 |  | 98 | 89 | -8.4 |  | 0 |
| Sep | 18.4 | 19.6 | 1.2 |  | 80 | 125 | 45.0 |  | 0 |
| Oct | 12.0 | 15.6 | 3.6 |  | 82 | 161 | 79.5 |  | 0 |
| CT-C & CT-S | | |  |  |  |  |  |  |  |
| Apr | 9.7 | 10.6 | 0.9 |  | 100 | 81 | -19.3 |  | 64 |
| May | 15.9 | 15.2 | -0.7 |  | 108 | 165 | 57.2 |  | 0 |
| Jun | 21.1 | 22.8 | 1.7 |  | 141 | 112 | -28.4 |  | 0 |
| Jul | 22.4 | 22.7 | 0.3 |  | 114 | 83 | -31.8 |  | 0 |
| Aug | 21.5 | 23.6 | 2.1 |  | 103 | 49 | -53.8 |  | 0 |
| Sep | 18.1 | 20.7 | 2.6 |  | 76 | 67 | -9.1 |  | 0 |
| Oct | 11.7 | 15.3 | 3.7 |  | 78 | 179 | 100.6 |  | 0 |
