# Supplementary material for: Field-scale evaluation of satellite-derived vegetation indices and image timing for in-season nitrogen management in corn
Source: Front Plant Sci. 2026 Mar 20;17:1731400. doi: 10.3389/fpls.2026.1731400 (PMC13047098; doi:10.3389/fpls.2026.1731400)
Supplement: Supplementary file 3 [file Table3.docx]

Supplementary Material

**Table S3.** Number of significant regression models (p < 0.10) and range of coefficients of determination (R²) and normalized RMSE (nRMSE) for relationships between vegetation indices (VIs) and grain yield across growth periods, field trials, and timings. Only significant models were included in the summary.

| Period | Field ^a^ | Timing ^b^ | Models | Significant models | min R^2^ | max R^2^ | min nRMSE | max nRMSE |
| --- | --- | --- | --- | --- | --- | --- | --- | --- |
| Veg | ALL | ALL | 144 | 85 | 0.02 | 0.29 | 0.15 | 0.24 |
|  | CT-C | ALL | 48 | 41 | 0.03 | 0.29 | 0.15 | 0.18 |
|  |  | 1 | 16 | 15 | 0.03 | 0.24 | 0.16 | 0.18 |
|  |  | 2 | 16 | 16 | 0.03 | 0.28 | 0.16 | 0.18 |
|  |  | 3 | 16 | 10 | 0.10 | 0.29 | 0.15 | 0.17 |
|  | CT-S | ALL | 48 | 25 | 0.02 | 0.26 | 0.18 | 0.20 |
|  |  | 1 | 16 | 3 | 0.03 | 0.05 | 0.20 | 0.20 |
|  |  | 2 | 16 | 9 | 0.02 | 0.17 | 0.19 | 0.20 |
|  |  | 3 | 16 | 13 | 0.02 | 0.26 | 0.18 | 0.20 |
|  | ST-S | ALL | 48 | 19 | 0.07 | 0.25 | 0.22 | 0.24 |
|  |  | 1 | 16 | 4 | 0.09 | 0.24 | 0.22 | 0.24 |
|  |  | 2 | 16 | 10 | 0.07 | 0.25 | 0.22 | 0.24 |
|  |  | 3 | 16 | 5 | 0.07 | 0.11 | 0.24 | 0.24 |
| Rep | ALL | ALL | 192 | 170 | 0.01 | 0.84 | 0.07 | 0.24 |
|  | CT-C | ALL | 64 | 59 | 0.04 | 0.84 | 0.07 | 0.18 |
|  |  | 4 | 16 | 12 | 0.04 | 0.28 | 0.16 | 0.18 |
|  |  | 5 | 16 | 15 | 0.07 | 0.49 | 0.13 | 0.18 |
|  |  | 6 | 16 | 16 | 0.31 | 0.84 | 0.07 | 0.15 |
|  |  | 7 | 16 | 16 | 0.36 | 0.78 | 0.09 | 0.15 |
|  | CT-S | ALL | 64 | 57 | 0.01 | 0.61 | 0.13 | 0.20 |
|  |  | 4 | 16 | 10 | 0.02 | 0.21 | 0.18 | 0.20 |
|  |  | 5 | 16 | 16 | 0.09 | 0.58 | 0.13 | 0.19 |
|  |  | 6 | 16 | 16 | 0.01 | 0.61 | 0.13 | 0.20 |
|  |  | 7 | 16 | 15 | 0.02 | 0.28 | 0.17 | 0.20 |
|  | ST-S | ALL | 64 | 54 | 0.13 | 0.81 | 0.11 | 0.24 |
|  |  | 4 | 16 | 11 | 0.14 | 0.70 | 0.14 | 0.24 |
|  |  | 5 | 16 | 16 | 0.13 | 0.81 | 0.11 | 0.24 |
|  |  | 6 | 16 | 15 | 0.23 | 0.70 | 0.14 | 0.22 |
|  |  | 7 | 16 | 12 | 0.23 | 0.61 | 0.16 | 0.22 |

*^a^ Field names indicate tillage (ST = strip-till; CT = conventional tillage) and previous crop (C = corn; S = soybean).*

*^b^ Timing: 1 (V7 and V8), 2 (V10 and V11), 3 (V15 and V16), 4 (R1 and R2), 5 (R3 and R4), 6 (R5 and R5.25), and 7 (R6).*
